# Experimental infection of Balb/c nude mice with Hepatitis E virus

**DOI:** 10.1186/1471-2334-9-93

**Published:** 2009-06-13

**Authors:** Fen Huang, Wen Zhang, Ga Gong, Congli Yuan, Yijia Yan, Shixing Yang, Li Cui, Jianguo Zhu, Zhibiao Yang, Xiuguo Hua

**Affiliations:** 1 Shanghai Key laboratory of Veterinary Biotechnology, School of Agriculture and Biology, Shanghai JiaoTong University, 800 Dongchuan Road, Shanghai 200240, PR China

## Abstract

**Background:**

Several animal species can reportedly act as reservoirs for Hepatitis E virus (HEV), a zoonotic pathogen. HEV and antibody to the virus have been detected in a variety of animals including rodents. Pig and rat models for HEV have been established for HEV, but a nude mouse has not yet been developed.

**Methods:**

Balb/c nude mice were inoculated with swine HEV, both orally and via intravenous injection to insure infection. Negative control and experimental contact-exposed groups of mice were also included in the study. The liver, spleen, kidney, jejunum, ileum, cecum and colon of each mouse from all three groups were collected for reverse transcription nested polymerase chain reaction (RT-nPCR) detection, indirect immunofluorescence observation and histopathologic examination. The sera from nude mice were tested for anti-HEV IgG by enzyme linked immunosorbent assay (ELISA). Activities of liver enzymes, including alanine aminotransferase (ALT), aspartate aminotransferase (AST) and alkaline phosphatase (ALP), as well as total bilirubin (TBIL) were also measured in the sera of the nude mice.

**Results:**

HEV antigens and HEV RNA were detected in liver, spleen, kidney, jejunum, ileum and colon both by indirect immunofluorescence and by RT-nPCR in all of the inoculated and in one of the contact-exposed nude mice. Histopathological changes were observed in the liver and spleen of these mice. Infected mice showed increased levels of AST, ALP, and anti-HEV IgG in sera. The livers of contact-exposed mice showed obvious histopathological damage.

**Conclusion:**

Nude mice could be readily infected by HEV isolated from pigs. The nude mouse may therefore be a useful animal model for studying the pathogenesis of HEV.

## Background

Hepatitis E (HE) is an acute self-limiting disease in adults that has particularly high mortality in pregnant women. The causative agent of HE, the HEV, is a zoonotic pathogen that is transmitted primarily by a fecal-oral route [1,2]. HEV shows cross-species transmission in pigs, chickens, rats, deer, cats and cattle [3-8]. Rodents are considered to be a potential reservoir of HEV because anti-HEV antibodies are widespread in both domestic and wild rats [9-13].

Some unsubstantiated evidence has even suggested that domestic rats may have been the original source of human HEV infection [11]. HEV has the ability to cross species barriers, causing infections between nonhuman primates and swine [14-19]. Therefore, from a human health standpoint, it is important to identify whether cross-species transmission is possible between swine and rodents.

Hepatitis E has widely been paid attention for antibody now found globally in the human population [7,20-22]. In developing countries, it is one of the most important causes of acute clinical hepatitis in adults [7]. Although cell culture systems for propagating hepatitis E have been developed [23-25], the availability of laboratory animal models is still of critical importance for studying HEV pathogenicity. The establishment of a nude mouse model for HEV infection could therefore possibly circumvent these issues. It would also have the added benefit of permitting the study of the molecular mechanisms of cross-species transmission, as well as the evaluation of vaccines. The aim of the present study was therefore to establish a HEV infection model in nude mice to aid in identifying the essential HEV transmission routes in this animal.

## Methods

### Animals

Twelve 5-week-old, 18~22 g specific-pathogen-free (SPF) male Balb/c nude mice were purchased from the National Rodent Laboratory Animal Resources, Shanghai Branch (China) and maintained in a pathogen-free animal facility. The study protocol was approved by Animal Care and Use Committee (ACUC) of Shanghai Research Center for Biomodel Organisms. We followed guidelines of the Shanghai Research Center for Biomodel Organisms during this study. All nude mice were tested for anti-HEV IgG by ELISA (KHB, Shanghai, China). Nude mice confirmed seronegative for HEV infection by ELISA were included in the study.

### Virus

The swine HEV for inoculation, characterized as genotype IV [GenBank: EF570133], was isolated from swine feces from the Shanghai area, China. The HEV RNA was detected by RT-nPCR [26,27]. Positive feces were suspended in phosphate-buffered saline (PBS), pH 7.4 with 0.01% diethyl pyrocarbonate (DEPC), at a proportion of 10% (w/v). The suspension was centrifuged at 12000 × g for 10 min, followed by filtration through 0.22 μm microfilters before inoculation. Virus was inoculated into each nude mouse at a minimum viral count of 1–2 × 10^5^/ml of feces supernatant, as calculated by viral genomic titer determined by Real-Time quantitative PCR [28,29].

### Experimental Design

Twelve SPF nude mice were randomly divided into three groups, four nude mice per group. Group No. 1 was the negative control intravenously inoculated with 0.25 ml sterilized PBS; Group No.2 was inoculated with swine HEV (0.25 ml intravenously and also 0.25 ml orally [29]); Group No.3 was used for experimental contact-exposure infection and consisted of three uninoculated SPF nude mice cohabiting with one inoculated nude mouse from Group No.2. Feces were collected daily post-inoculation. Each nude mouse was humanely euthanized, at either 4, 7, 14 or 21 days post-inoculation following the guidelines of the Care and Use of Laboratory Animals. Blood was collected for RT-nPCR detection, ELISA tests and enzyme activity assays. Liver, spleen, kidney, jejunum, ileum, cecum, and colon were collected, and each tissue was divided into three portions for RT-nPCR detection, indirect immunofluorescence observation and histopathologic examination, respectively. Two portions were stored at -80°C until use, while the third portion was fixed in 10% neutral buffered formalin for histopathologic examination immediately upon sampling.

### ELISA determination

HEV IgG was determined using a commercial ELISA kit (KHB, Shanghai, China) based on recombinant HEV fusion proteins according to the manufacturer's directions. The kit used recombinant HEV fusion proteins derived from the putative structural proteins of HEV as primary antibody, and contained both positive and negative controls. The cutoff values for the IgG assay were determined based on 0.22 plus the mean OD_450 _values of sera from uninfected nude mice (± standard deviation).

### Serum liver chemistry profile

The activities of ALT, AST, ALP and levels of TBIL in sera were measured with an automated biochemistry analyzer (Olympus 2700, Japan).

### RT-PCR detection

Total RNA was extracted from all specimens by Trizol (Invitrogen, America), according to the manufacturer's instructions. Reverse transcription was performed using an AMV Reverse Transcriptase XL for RT-PCR (Takara, Japan) according to the manufacturer's directions. HEV-specific primers have been previously described [26]; the external primers were forward primer (P1): 5'-AATTATGCC(T)CAGTAC(T)CGG(A)GTTG-3' and reverse primer (P2): 5'-CCCTTA(G)TCC(T)TGCTGA(C)GCATTCTC-3', and the internal primers were forward primer (P3): 5'-GTT(A)ATGCTT(C)TGCATA(T)CATGGCT-3' and reverse primer (P4): 5'-AGCCGACGAAATCAATTCTGTC-3'. The product of RT-nPCR was expected to be 348 base pairs. The RT-PCR protocol was performed at 30°C for 10 min, 42°C for 30 min, 99°C for 5 min and 5°C for 5 min. The resulting cDNA was amplified by nested PCR at 94°C for 2 min, followed by 94°C for 30 sec, 42°C for 30 sec and 72°C for 1 min, and repeated for 29 cycles. The PCR products were detected by electrophoresis on agarose gel containing 0.5 μg/ml ethidium bromide.

### Indirect immunofluorescence observation

Frozen tissues were cut at a thickness of 6 μm for indirect immunofluorescence observation. HEV-specific ORF2 primary antibody (ABR, America, 1:500 dilution), designed to detect capsid proteins of HEV genotypes (1–4), was added to sections and incubated at 37°C for 30 min. After washing with PBS, FITC-labelled goat anti-mouse secondary antibody (Dingguo, China, 1:500 dilution) was added and incubated at 37°C for 30 min. After washing with PBS, slides were observed with a Nikon TE2000 fluorescence microscope (Japan). All specimens were tested in duplicate with positive control (specimens of HEV infected rat) and negative control (specimens of uninfected nude mice).

### Histopathologic examination

Tissues for histologic examination were fixed in 10% neutral buffered formalin, routinely processed, sectioned at a thickness of 7 μm, and stained with hematoxylin and eosin. The tissue sections were examined and compared with negative controls.

## Results

### Clinical evaluation

Evidence of clinical disease, such as acute hepatitis with icteric viral hepatitis or diarrhea in humans, was not found in any of the experimental groups.

### Detection of HEV RNA by RT-nPCR

HEV RNA was detected by RT-nPCR, beginning four days after inoculation in feces from all inoculated nude mice. In livers, spleens, kidneys, jejunums, ileums and colons, RNA was detected 4, 7 and 14 days post-inoculation, and in sera on days 4 and 7 post-inoculation. HEV RNA was detected in the feces of only one of the contact-exposed mice from day 7 to 20 post-inoculation, but was found in liver, spleen, kidney and sera sampled on day 14. However, HEV RNA was not detected in any of the tissues, sera or feces in the negative controls.

### ELISA determination

**Figure 1:**
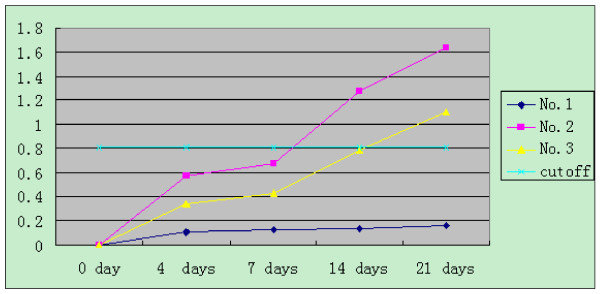
**Anti-HEV IgG in nude mice as determined by ELISA**. Anti-HEV IgG was significantly elevated in the inoculated group (No.2, pink line) but increases were slower to appear in the contact-exposed group (No.3, yellow line). No changes were seen in the negative control group (No.1, dark line). The cutoff is 0.22+ the mean values of the negative control (blue line).

An increased anti-HEV IgG was seen in all inoculated mice compared with the negative control group. Although IgG was also elevated in the contact-exposured group, but the increase was slower to appear and lower in magnitude than that seen in the inoculated group. All assays were performed in triplicate and data are expressed as means (± standard deviation). The mean values of OD_450 _for the three groups were analyzed by SAS System software [Figure 1].

### Liver enzyme profile

**Figure 2:**
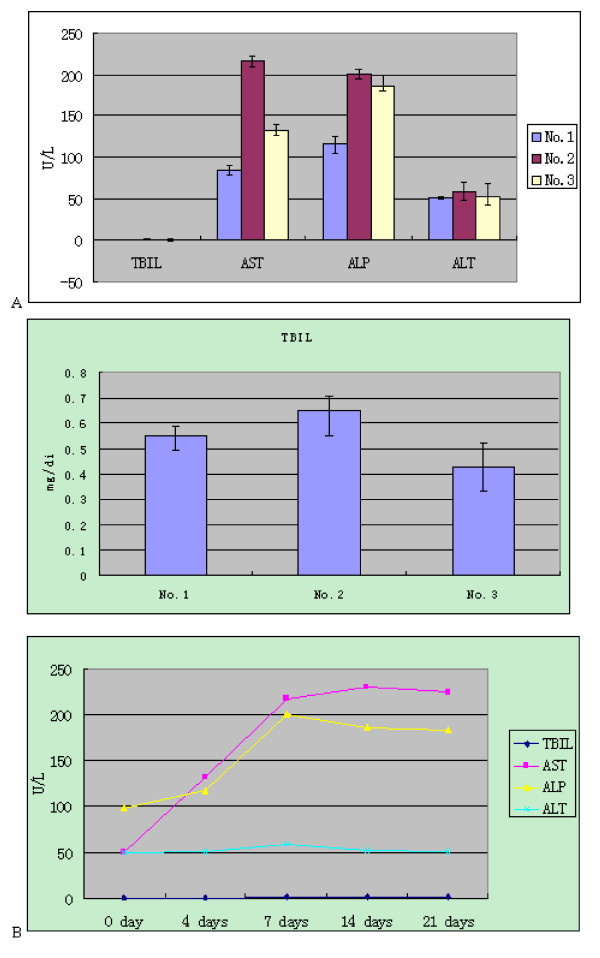
**Changes in enzyme activities for AST, ALP, ALT and in levels of TBIL**. A: The levels of AST, ALP, ALT activities and TBIL in the uninoculated group, the inoculated group and the contact-exposed group 7 days post-inoculation. The data represent the average of three experiments, with the standard deviation indicated by error bars. B: Enzyme activity changes in nude mouse in three groups.

Liver enzyme activities were characterized using an automated biochemistry analyzer. There were no significant differences in the levels of TBIL or ALT among the three groups. The level of AST was increased (about 2.6 fold) in the inoculated group (No.2) and moderately increased in contact-exposed group (No.3) compared with the negative control group (No.1). The level of ALP was increased in both the inoculated group (No.2) and the contact-exposed group (No.3) [Figure 2A]. All procedures were performed in triplicate and data are expressed as means (± S.D.). The changes in the three enzyme activities and in bilirubin levels (AST, ALT, ALP, and TBIL) among three groups are shown in [Figure 2B].

### Indirect immunofluorescence

**Figure 3:**
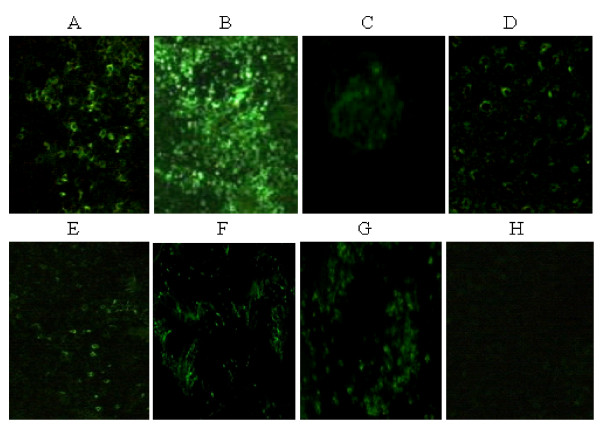
**HEV antigen distribution in tissues of inoculated nude mice detected by indirect immunofluorescence 4d post-inoculation**. A: liver, granular fluorescence of HEV antigens distributed throughout hepatocytes, × 200; B: spleen, distinct granular fluorescence of HEV antigens distributed throughout the cytoplasm, especially around the venous sinus, × 100; C: kidney, dozens of HEV antigens surrounding a renal cell, × 200; D: jejunum, distinct granular fluorescence of HEV antigens distributed in the epithelial cells, × 200; E: ileum, granular fluorescence of HEV antigens distributed in the ileum, × 200; F: colon, distinct granular fluorescence of HEV antigens distributed throughout colon, × 100; G: positive control, liver of HEV infected rat, × 100; H: negative control, liver of a nude mouse from the negative control group, × 200.

HEV antigens were observed by indirect immunofluorescence in liver, spleen, kidney, jejunum, ileum and colon in the inoculated group on days 4 and 7, and in one of member of contact-exposed group on day 7 using appropriate controls [Figure 3A–F]. HEV antigen was consistently detected in the cytoplasm [Figure 3A], splenocytes [Figure 3B] and renal cells [Figure 3C], and interspersed in jejunum [Figure 3D], ileum [Figure 3E] and colon [Figure 3F] in all the inoculated mice and in one of the contact-exposed mice. The HEV antigen was abundant in the liver and especially in the spleen. We also identified several potential HEV replication sites in jejunum, ileum and colon that have not been reported in nude mice prior to this study. As we expected, one of the contact-exposed mice showed HEV antigens in spleen, liver, and colon, which is consistent with previous studies following contact exposure transmission [11,13,30]. We found no signal in any of the negative control tissues [Figure 3H].

### Histopathologic examination

**Figure 4:**
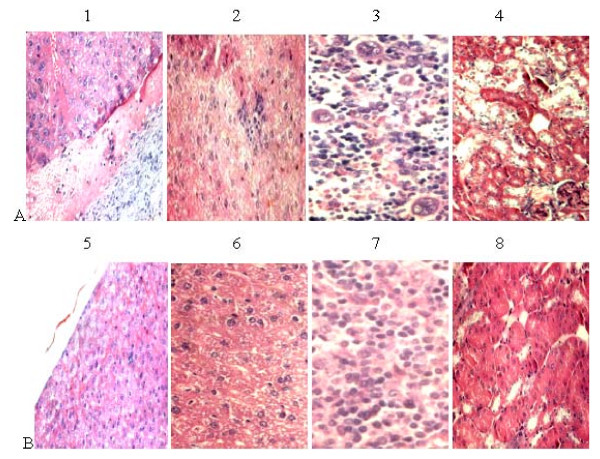
**Histopathologic changes in the inoculated group on day 21**. A: 1, liver, one of the inoculated nude mice showed an enlarged liver filled with inflammatory exudates, × 200; 2, liver, severe lymphoplasmacytic and histiocytic hepatitis, focal accumulation of inflammatory cells encircling hepatocytes, × 200; 3, spleen, the ruptured and enlarged spleen displaying multiple vacuolar degeneration and focal necrosis, × 200; 4, kidney, the disarranged kidney cells with increased lymphocytes and cellular debris, × 200 B: 5, negative control liver, × 200; 6, negative control liver, × 200; 7, negative control spleen, × 200; 8, negative control kidney, × 200. Tissues were stained with hematoxylin and eosin.

Histopathologic examination of the livers showed enlargement in one of inoculated mice, with liver capsules filled with inflammatory exudates and liver hemorrhage [Figure 4-A-1]. Hepatic inflammation, focal hepatocelluar necrosis and liver hemorrhage were observed in all inoculated mice, as well as in the contact-exposed group [Figure 4-A-2]. The hepatic lesions were mild in contact-exposed mice but severe in the inoculated ones. Lymphocytic, necrotic and cellular debris was observed in the spleens of inoculated mice and in milder form in contact-exposed mice [Figure 4-A-3]. Increased expression of infiltrating lymphocytes and macrophages in renal cells were observed [Figure 4-A-4]. No damage was observed in any of the negative control tissues [Figure 4-B 5 to 4-B 8]. The biochemical, histologic, virologic and serologic changes caused by HEV inoculation and/or contact exposure is briefly summarized in Table 1.

**Table 1:** Biochemical, histologic, virologic and serologic changes in response to experimental inoculation of nude mice with HEV

Group		No.1	No.2	No.3
HEV-RNA (feces)	0d	-	-	-
	4d	-	+(4/4)	-
	7d	-	+(4/4)	+(1/4)
	14d	-	+(4/4)	+(1/4)
	21d	-	-	-
	Liver	-	+(4/4)	+(1/4)
	Spleen	-	+(4/4)	+(1/4)
	Kidney	-	+(4/4)	+(1/4)
HEV-antigen	Jejunum	-	+(4/4)	-
	Ileum	-	+(4/4)	-
	Colon	-	+(4/4)	-
Anti-HEV IgG		-	+(4/4)	+(1/4)
IHC		-	+(4/4)	+(1/4)
ALT		---	---	---
AST		---	↑	↑
ALP		---	↑	↑
TBIL		---	---	---
Histologic change		no change	obvious	obvious

-: HEV RNA negative detected by RT-PCR; +: HEV RNA positive detected by RT-PCR; IgG: the positive proportions of HEV antibodies detected by ELISA 21 days post-inoculation; IHC: the positive proportions of HEV antigen detected by indirect immunofluorescence; ---: no significant changes; ↑: significantly elevated compared with a negative control.

## Discussion

The results presented here strongly suggest that nude mice are susceptible to swine HEV as evidenced by viral antigen expression in liver and other extra-hepatic tissues, fecal viral shedding, hepatic lesions, and the presence of anti-HEV antibodies. The inoculated mice showed no clinical signs of HEV infection, as has been previously reported for other animal models, such as pigs and rats [14,10,31,11,18]. These findings reported here are helpful for the study of the replication mechanism, transmission and pathogenesis of HEV.

We first detected HEV RNA in the feces 4 days post-inoculation in agreement with previous findings in rats (4 day) [11], cynomolgus macaques (4–6 day) [14] and pigs (7 day) [32]. In one of the contact-exposed nude mice, HEV RNA was first detected 7 days post-inoculation; i.e., 3 days later than in the directly inoculated mice. This delay reflected the lag time for virus to appear in the feces of the cohabiting inoculated mouse, and indicates that contact exposure infection occurred primarily by a fecal-oral route. In the liver, spleen, kidney, and colon of inoculated mice, HEV RNA was detected by RT-nPCR on day 4, 7, and 14, and HEV antigens were observed by indirect immunofluorescence. A large number of fluorescent of HEV antigen were observed in liver, spleen, and kidney, indicating these to be heavily infected tissues. Only a few suspected fluorescent HEV antigens were seen in the jejunum, ileum and colon [Figure 3]. This would indicate that HEV was probably replicating in these tissues, which is consistent with the previous studies [33], but this need to be further demonstrated with *in situ *hybridization. We detected HEV RNA in sera on day 4 and 7, but were unable to perform daily serum collection. In nonhuman primates and swine, the HEV RNA can be detected in bile [33], but it is not possible to obtain bile from a nude mouse owing to its small body size.

HEV infection in rats has been shown to have some aspects in common with HEV infection in nonhuman primates and swine; but one exception was that levels of ALT liver enzymes did not become elevated [11]. In the current study, ALT levels in nude mice were also unchanged by HEV infection in nude mice. Aggarwal et al (2001) has confirmed that ALT elevation may not a useful indicator of HEV infection in primates [20]. In contrast, the level of AST was significantly elevated in inoculated (2.6 fold) and contact exposed nude mice (1.6 fold). In addition, the sera of inoculated and contact exposed mice had elevated ALP activities, but no increases in TBIL were seen in response to HEV infection over the 21 days duration of the study.

The presence of HEV in stool can serve as a major source of transmission by contact exposure to other animals. In the current study, one of the contact-exposed mice became infected from viruses excreted by an inoculated nude mouse. This contact-exposed mouse also showed similar results to those shown by the inoculated nude mice, except that the anti-HEV antibody response was slower and AST liver enzyme levels were not as elevated. These findings are in agreement with a previous report of contact exposure in swine [13]. Taken together, the data presented here show that HEV from swine can infect nude mice and that this infection can readily occur through fecal-oral transmission.

## Conclusion

HEV replication mechanisms, cross-species transmission and transmission by contact exposure could all be examined following experimental infection of Balb/c nude mice with swine HEV. Therefore the nude mouse could be considered as a promising animal model for HEV studies, especially to evaluate candidate vaccines or antiviral treatments.

## Competing interests

The authors declare that they have no competing interests.

## Authors' contributions

All authors participated in the planning of the project. XH was the leader of the project. FH performed the nude mice breeding and the main experiments. WZ, GG, CL, YJ carried out the IIF and ELISA determinations together with FH. SX collected all of the samples. All authors read and approved the final manuscript.

## Pre-publication history

The pre-publication history for this paper can be accessed here:

http://www.biomedcentral.com/1471-2334/9/93/prepub
